# Change blindness in simulated driving in individuals with homonymous visual field loss

**DOI:** 10.1186/s41235-022-00394-6

**Published:** 2022-05-15

**Authors:** Garrett Swan, Jing Xu, Vilte Baliutaviciute, Alex Bowers

**Affiliations:** 1 Schepens Eye Research Institute of Massachusetts Eye and Ear, Department of Ophthalmology, Harvard Medical School, 20 Staniford St., Boston, MA 02114 USA; 2 Envision Research Institute, Wichita, KS USA

## Abstract

Individuals with homonymous visual field loss (HVFL) fail to perceive visual information that falls within the blind portions of their visual field. This places additional burden on memory to represent information in their blind visual field, which may make visual changes in the scene more difficult to detect. Failing to detect changes could have serious implications in the context of driving. A change blindness driving simulator experiment was conducted with individuals with HVFL (*n* = 17) and in those with normal vision (NV; *n* = 16) where changes (pedestrians appearing) were triggered based on the driver’s gaze location. Gaze was used to ensure that the location of the change was visible before and after the change occurred. There were wide individual differences in both vision groups, ranging from no change blindness to more than 33% of events. Those with HVFL had more change blindness than those with NV (16.7% vs. 6.3%, *p* < 0.001) and more change blindness to pedestrians appearing in their blind than seeing hemifield (34.6% vs. 10.4%, *p* < 0.001). Further, there was more change blindness for events appearing in the seeing hemifield for those with HVFL than normal vision (*p* = 0.023). These results suggest that individuals with HVFL may be more susceptible to failures of awareness, such as change blindness, than individuals with normal vision. Increased risk for failures of awareness may result in motor vehicle crashes where the driver fails to notice the other road user (looked-but-failed-to-see incidents).

## Significance statement

A common response following a motor vehicle accident is that the driver “looked but failed to see” the collision object. One proposed mechanism for these incidents is change blindness, the failure to detect a salient change following a visual disruption. While studies have found change blindness in laboratory-based and simulator paradigms using visual disruptions (flickers) or between repeated drives, we developed a more realistic, novel paradigm in a driving simulator where changes were gaze-triggered, such that they happened while the driver was looking toward the other side of the intersection. We investigated whether individuals with homonymous visual field loss would be more susceptible to change blindness, given that the lack of peripheral vision places additional burden on memory to represent information in the blind hemifield. We found change blindness in both normally sighted individuals and those with homonymous visual field loss, with wide individual differences in both groups. These results suggest that some hazard detection failures when driving may be veridical failures of visual awareness and that our methodology may be a viable approach to study change blindness in realistic driving situations. Furthermore, we found evidence that those with visual field loss were more likely to have change blindness than those with normal vision, suggesting that even if they compensate sufficiently by scanning, they may still be more prone to hazard detection failures from failures of visual awareness than drivers with normal vision.

## Introduction

“Looked but failed to see” (LBFTS) incidents are motor vehicle collisions where the driver reports looking, but fails to see the collision object (Treat, 1980; Stutts et al., 2003; Koustanaï et al., 2008). Multiple mechanisms have been proposed to account for such incidents, including change blindness, which is the failure to detect a salient change when that change occurs during a brief disruption (Jensen et al., 2011). For a LBFTS incident to be true change blindness, the driver needs to look toward the change location both before and after the change. For example, if a driver collided with a pedestrian that suddenly appeared from behind a parked car and the driver had looked at the parked car before and after the pedestrian appeared, then this could potentially be a true change blindness event. However, if the driver looked nowhere near the parked car before the pedestrian appeared, then failure to detect the pedestrian would not be change blindness, but some other kind of failure of awareness (e.g., inattentional blindness). Without any objective measures of gaze behaviors, it is impossible for crash investigators to determine whether a true LBFTS incident occurred and whether change blindness might have been the underlying cause. For these reasons, laboratory and simulated driving tasks have been widely used to investigate change blindness as a mechanism underpinning failures of awareness when driving.

Researchers have shown change blindness to hazards using laboratory tasks, such as the flicker paradigm (Caird et al., 2005; Galpin et al., 2009; Koustanaï et al., 2012; McCarley et al., 2001, 2004), with potential hazards being easier to detect than non-hazards (Beanland et al., 2017). In driving simulation, some researchers have inserted changes between drives, such as a speed limit sign that changes (Harms & Brookhuis, 2016) or other objects (e.g., buildings) in the environment (Charlton & Starkey, 2013). However, those paradigms do not address failures of awareness that may happen within a driving scene, such as when a pedestrian suddenly appears from behind a car. Many studies have used a flicker paradigm with inserted blanks, while participants drove in a simulator (Velichkovsky et al., 2002; Lee et al., 2007; Filtness et al., 2020; White & Caird, 2010). The artificial blanks represented the type of global disruptions caused by eye movements or a blink (Simons, 1996). This approach is limited in ecological validity because the blanks are simulations of the visual disruptions, which might or might not have occurred at the time of an eye movement. In addition, eye movements have not always been tracked, so it is not always known whether all the conditions for change blindness were met (e.g., Lee et al., 2007; Filtness et al., 2020), and if eye tracking was recorded, it can be rare for a change to occur during an eye movement serendipitously (Velichkovsky et al., 2002).

Here, we developed a driving simulator paradigm with gaze-triggered changes to investigate change blindness to potential hazards while driving. Our goal was to address some of the limitations of prior methodology. Our novel paradigm simulated a real-world situation where change blindness may occur, i.e., a pedestrian suddenly appearing when looking at the other side of an intersection. Instead of using an artificial disruption while driving (e.g., a flicker, Zhang & McConkie, 2010), changes were inserted during a natural disruption in vision when the participant was looking away from the location of the change. When participants scanned toward the other side of the intersection and fixated on cross-traffic, a pedestrian appeared on the opposite side of the intersection. This ensured that changes occurred in the periphery. Then, we used the gaze data to verify that the location of the change was visible before and after being triggered.

One population of individuals who may be more susceptible to change blindness are those with homonymous visual field loss (HVFL), which is a loss of vision in the same parts of the visual field in both eyes caused by lesions to the postchiasmal visual pathways following stroke or traumatic brain injury (Goodwin, 2014; Zhang et al., 2006). In 2018, there were 7.8 million stroke survivors in the USA (Villarroel et al., 2019), with up to 60% experiencing a visual impairment following the stroke (Rowe et al., 2019; Zhang et al., 2006). In the case of homonymous hemianopia, these individuals are blind (unable to see) in half of their visual field (i.e., left or right), which may be permanent in approximately 10% (Zhang et al., 2006) to 30% (Townend et al., 2007) of cases. Recovery of visual field following 3 months is rare (Gray et al., 1989). Individuals with HVFL are permitted to drive in jurisdictions of the USA where they meet the horizontal visual field requirements (e.g., New Hampshire has no field requirements) (Peli, 2002) as well as some other countries (e.g., Belgium, the Netherlands, Switzerland, Canada, and the UK have conditional licenses). Moreover, some individuals continue to drive despite not legally meeting the horizontal visual field requirement (Bowers et al., 2009).

Individuals with HVFL can compensate for their visual field loss by scanning toward their blind visual field (Gassel & Williams, 1963), but need to scan at least as far as the object of interest in order to see it, which is critical when they drive. In driving studies, inadequate scanning (not scanning far enough into the blind field to fixate the object of interest) is a common reason for failing to detect objects in the blind hemifield (Bahnemann et al., 2015; Bowers et al., 2014; Kübler et al., 2015; Swan et al., 2021). However, analysis of gaze tracking data suggested that some of the blind hemifield detection failures of individuals with HVFL in a driving simulator study may have resulted from a failure of visual awareness (Bowers et al., 2015). Specifically, in a few events, participants with HVFL scanned far enough toward the blind hemifield to fixate a pedestrian that initially appeared in the blind hemifield but still failed to respond to it. Similarly, in a laboratory-based flicker paradigm, those with HVFL experienced more change blindness than those with normal vision (Muir, 2017). People with HVFL may be more prone to failures of visual awareness than people with normal vision (NV) because there are additional demands placed on memory to represent information in the blind portion of the visual field and they may experience more profound disruptions in vision during scanning than those with NV. We therefore implemented our novel driving simulator change blindness paradigm to test the hypothesis that those with HVFL would have higher rates of change blindness than those with normal visual fields.

## Methods

### Participants

Twenty individuals (Table 1) with HVFL and 17 age similar NV controls were recruited to participate in the experiment. All participants had good visual acuity (Table 1). For the individuals with HVFL, their visual fields were plotted using a Goldmann perimeter to verify that they had HVFL. Furthermore, the participants with HVFL were tested for spatial neglect, which was measured with both the Schenkenberg line bisection test (Schenkenberg et al., 1980; Van Deusen, 1988) and the Bells test (Vanier et al., 1990). None of the participants had spatial neglect. Finally, cognitive status of participants with HVFL was evaluated with the Montreal Cognitive Assessment (MoCA; Nasreddine et al., 2005). One participant with HVFL was excluded because they had a MoCA score of 12, suggesting cognitive impairment. Three participants (2 HVFL, 1 NV) were omitted from the analyses because they failed to understand the instructions of the experiment. Of the 17 participants that qualified in the HVFL group, 13 had complete homonymous hemianopia (9 left and 4 right), 1 had upper left quadrantanopia, and 3 had lower left quadrantanopia. The majority of participants with HVFL were not current drivers since individuals with hemianopia are not permitted to drive in Massachusetts where the study was conducted. However, they all wanted to drive, if permitted. The study followed the tenets of the Declaration of Helsinki and was approved by the institutional review board at the Schepens Eye Research Institute.

**Table 1:** Demographic information for those with homonymous visual field loss (HVFL) and normal vision (NV)

	HVFL, *n* = 17	NV, *n* = 16	*p* value
Current driver, n (%)	3 (17.7%)	14 (87.5%)	< 0.001
Male, n (%)	14 (82%)	10 (62.5%)	0.26
Age, y, median (IQR)	50.0 (32.8)	49.5 (32.5)	0.54
Race, n (%) reported White	15 (88.2%)	9 (56.3%)	0.06
Visual Acuity (LogMAR), mean (SD) Snellen equivalent	− 0.06 (0.1) 20/17	− 0.05 (0.09) 20/18	0.67
Left HVFL, n (%)	13 (76%)	NA	NA
MoCA score, mean (SD)	26.9 (2.5)	NA	NA
Hemianopia caused by stroke, n (%)	10 (58.8%)	NA	NA
Years since onset, median (IQR)	3.0 (7.5)	NA	NA

*LogMAR* Logarithm of the Minimum Angle of Resolution

*MoCA* Montreal Cognitive Assessment (Nasreddine et al., 2005)

*p* Values for between group differences were calculated using Fisher exact test for categorical variables and Mann–Whitney *U* test for continuous variables

### Apparatus

**Fig. 1:**
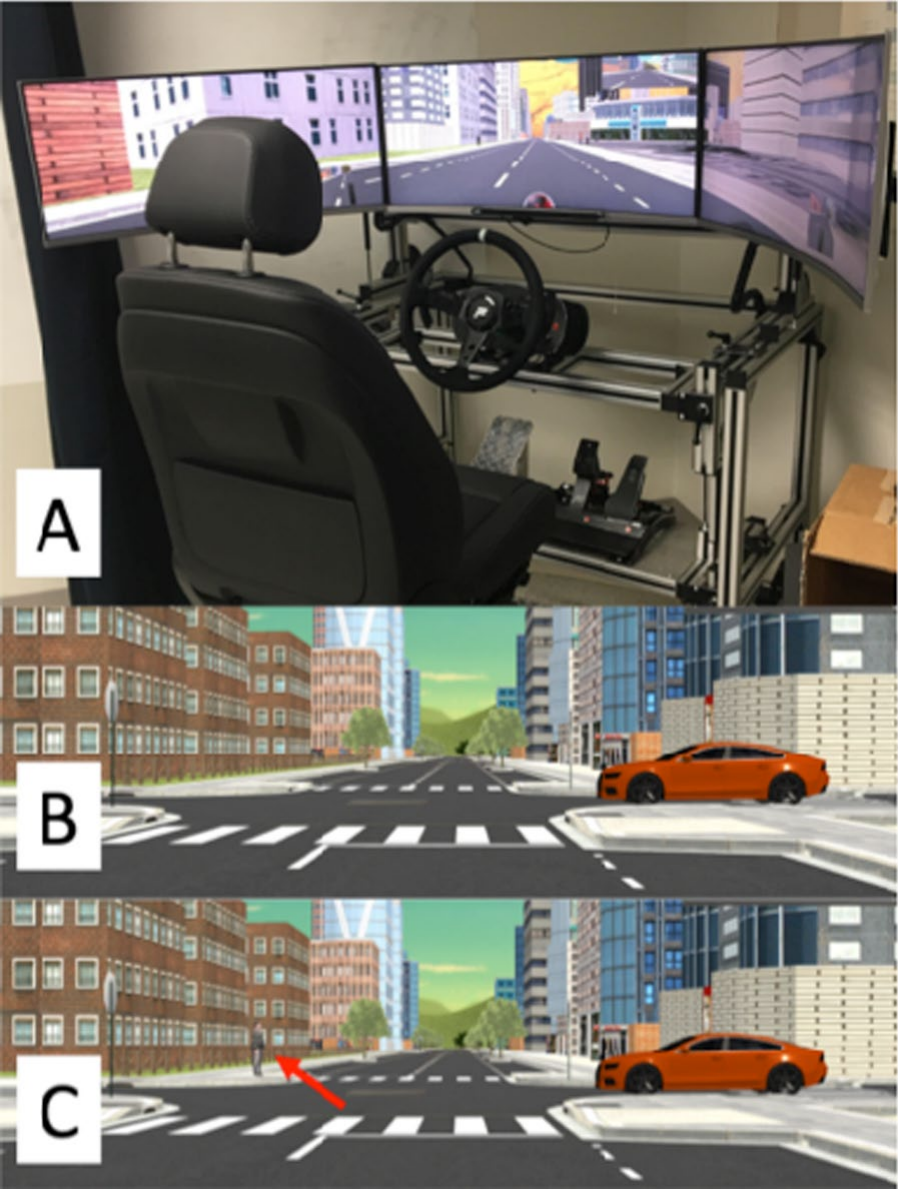
Images of the driving simulator and virtual world. **A** The driving simulator. **B** The center screen showing an intersection without a pedestrian and **C** The center screen showing a life-size pedestrian (see red arrow) appearing near to the curb as in condition A (see Fig. 2) from a static viewpoint. The car in both (**B**) and (**C**) on the right of the intersection is the cross-traffic on which the participant had to fixate in order to trigger the appearance of the pedestrian (see Fig. 3, bottom panel)

Participants drove in a custom-built driving simulator (Lehsing et al., 2019) in a dimly lit room at Schepens Eye Research Institute (Fig. 1). The simulator displayed the virtual world on 3 screens that provided 165.5° horizontal and 26.5° vertical field of view (Samsung CF791, 34″ with 21:9 widescreen ratio, 3440 × 1440 resolution). Prior to initiating the experiment, participants were allowed to adjust the distance of the steering wheel, accelerator and brake, and the orientation of the seat. Rear view mirrors and a speedometer were displayed on the screens. Participants used a response button on the steering wheel to indicate noticing of a change. A response button was located on both sides of the steering wheel, and participants were encouraged to use either.

While driving, the participant’s gaze was recorded with a Tobii 4C tracker (Tobii Eye Tracker 4C, Sweden) at 50 Hz. The Tobii 4C was placed at the bottom of the center screen and recorded the position of gaze as onscreen coordinates. Prior to the experimental drives, gaze was calibrated with Tobii’s 6-point calibration software and validated using custom-built software in Unity (9-point verification). In cases where the 6-point calibration was not locating gaze, then we adjusted the distance of the participant from the gaze tracker by moving the seat forward or back. The gaze tracking data were synced with the driving simulator data through Unity.

### Stimuli

**Fig. 2:**
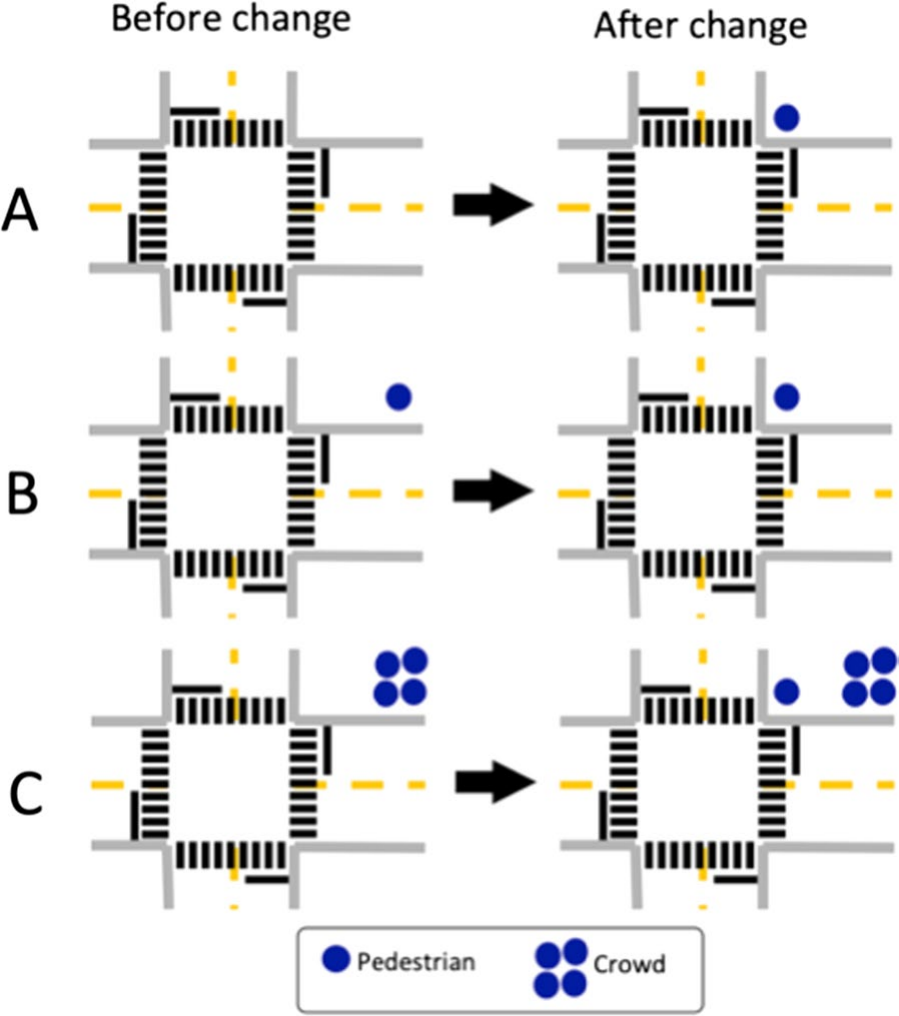
Illustrations of the different conditions. **A** There is no pedestrian at the intersection, and then, a critical pedestrian appears near the crosswalk. **B** There is a pedestrian far from the crosswalk that disappears simultaneously as the critical pedestrian appears near the crosswalk. **C** There is a crowd far from the crosswalk, and the critical pedestrian appears near the crosswalk. In all three conditions, the critical pedestrian appeared in the same location. These conditions were also used for the distance-triggered pedestrians

The simulated driving environment and stimuli were created and presented in Unity (version 2017.4.40f1). Pedestrian scenarios were implemented along two unique routes (each with 45 intersections) through a custom-built city with roads on a grid system. Auditory GPS-like navigation (“turn left at next intersection”) guided participants through the routes. The roads were lined by trees and buildings and had oncoming and cross-traffic. All pedestrians were stationary and appeared as a single pedestrian or a crowd (3 to 8). They varied in clothes and sex/gender and were approximately 1.8 m tall. Pedestrians for the critical events (section "critical events") appeared at intersections where participants drove straight through. They appeared in two possible locations: near the crosswalk (1.5 m from edge, Figs. 1 and 2) or far from the crosswalk (6.5 m from edge, Fig. 2). The crowds of pedestrians only appeared in the location further away from the crosswalk. In addition, pedestrians and crowds were placed in various other locations throughout the virtual world to reduce the likelihood of predicting critical pedestrians.

### Critical events

#### Gaze-triggered events

**Fig. 3:**
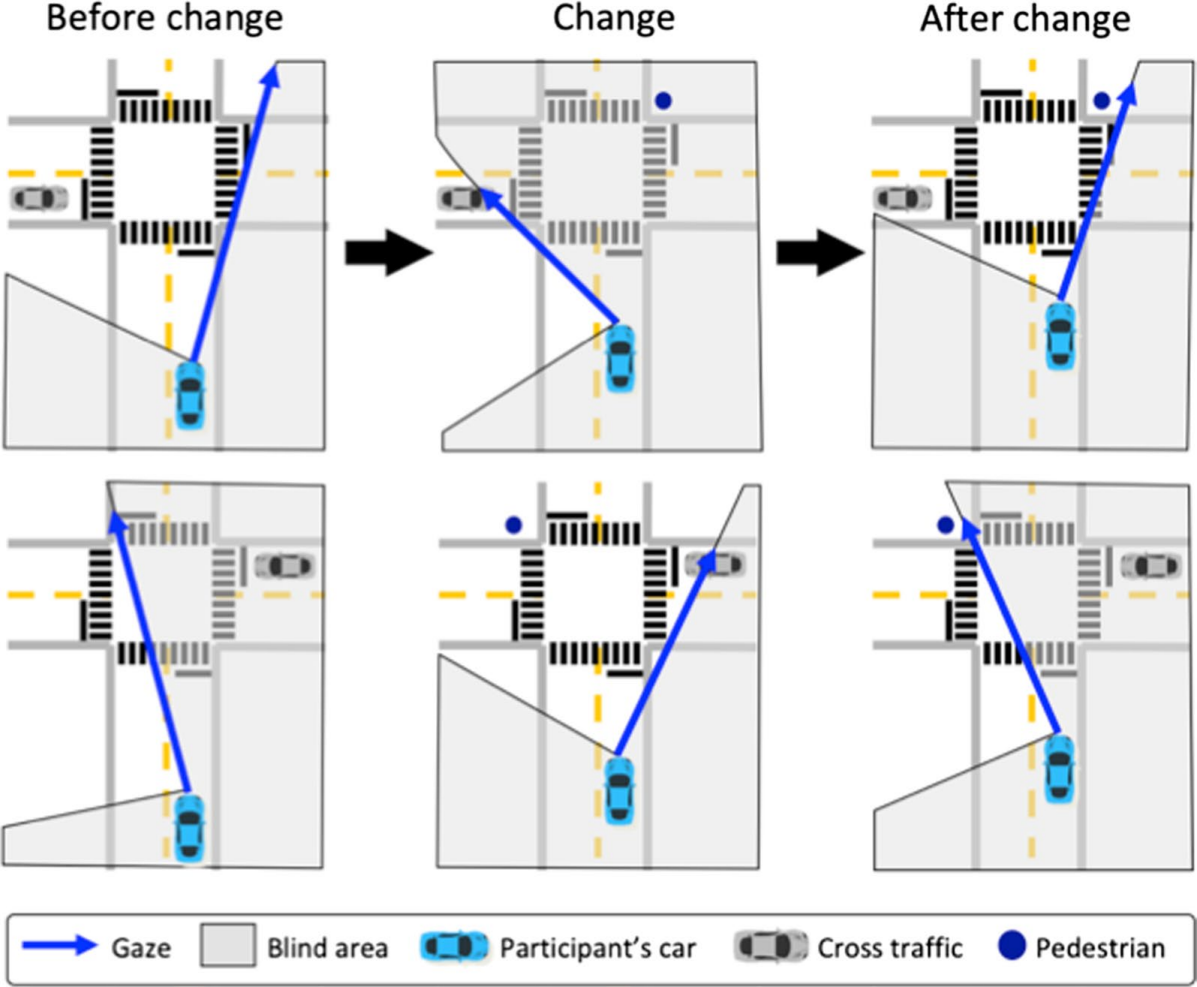
Illustrations of the gaze-triggered critical pedestrians in condition A in a participant with right HVFL. In the top middle panel, the pedestrian appears in the blind hemifield, and in the bottom middle panel, the pedestrian appears in the seeing hemifield. In both examples, the location of the change was visible before the change in the panels on the left. Then, in the next panels (middle), the participant fixates on the cross-traffic, which triggers the sudden appearance of the pedestrian. In the panels on the right, the participant looks back and the pedestrian is visible in the seeing hemifield. A failed detection of the change would be categorized as change blindness

For the gaze-triggered events, a critical pedestrian appeared if and only if the participant looked at the cross-traffic that appeared on the opposite side of the intersection from the pedestrian (Fig. 3). Cross-traffic moved at 56.3 km/h (35 mph) toward the intersection once the participant’s vehicle was within 88.5 m of the white line of the intersection and then stopped prior to entering the intersection. If the participant looked at the cross-traffic, the critical pedestrian appeared near the crosswalk. We had three possible conditions to minimize predictability (Fig. 2). The critical pedestrian could be paired with (A) no other pedestrian, (B) a pedestrian at the far location from the crosswalk that disappeared once the critical pedestrian appeared, or (C) a crowd of pedestrians at the far location from the crosswalk. There were 12 total gaze-triggered events per drive, with equal numbers on the left and right and equal numbers of conditions.

#### Distance-triggered events

Distance-triggered events were included in case participants did not scan sufficiently to trigger the gaze-triggered events. Distance-triggered pedestrians were the same as the gaze-triggered pedestrians except that the pedestrian appeared once the driver was 40 m from the white line of the intersection. A 4th distance-triggered event that was the same as condition B except in reverse (i.e., pedestrian moved from the near location to the far location) was also used to reduce predictability and not included in the analyses. There were 8 total distance-triggered events per drive, with equal numbers on the left and right and equal numbers of pairings.

#### Catch events

We programmed catch events to minimize predictability of when and where critical pedestrians would appear. These events consisted of pedestrians that were located near the crosswalk without cross-traffic, crowds located far from the crosswalk without cross-traffic, cross-traffic without any pedestrians, and some intersections without cross-traffic or pedestrians.

### Procedure

Participants completed a series of practice drives before the experimental drives. The first practice drive took place in the same city as the experimental drives and involved practicing vehicle control skills. Given that some participants were not current drivers, they were all required to demonstrate sufficiently good vehicle control before starting the second practice drive, which involved all of the components found in the experimental drives. Participants were asked to follow auditory GPS-like navigation instructions, stop at the appropriate signage, and press the response button whenever they noticed that a pedestrian had suddenly appeared. At the beginning of the second practice drive, there were 4 examples of the pedestrian appearing with the experimenter explaining and pointing out the behavior of the pedestrians. After the 4 examples, there were 8 additional examples that the experimenter observed and provided feedback (if necessary) to facilitate understanding of the task.

Following the practice drive, participants completed 2 experimental drives that were counterbalanced between participants. Each drive took between 8 and 12 min.

### Data processing

Events were processed using custom MATLAB (MathWorks, R2014b) scripts with the goal of (1) identifying if the change occurred, (2) determining if that change occurred while the driver was looking at the other side of the intersection, (3) determining if the location of the change was visible before and after the change, and (4) categorizing the event as detection if the driver noticed the change or change blindness if the driver did not notice the change. First, we determined whether the change occurred, while the participant was looking toward the opposite side of the intersection by comparing the sign of gaze eccentricity to the sign of the eccentricity of the changed pedestrian, with changes on the left side of the intersection being −1 and changes on the right side being 1. Eccentricities to the left of the straight-ahead position (i.e., 0°) were processed as negative values. Gaze-triggered events that met this criterion and distance-triggered events where gaze was serendipitously on the opposite side of the intersection when the critical pedestrian appeared were included in analyses; all other distance-triggered events were excluded.

**Fig. 4:**
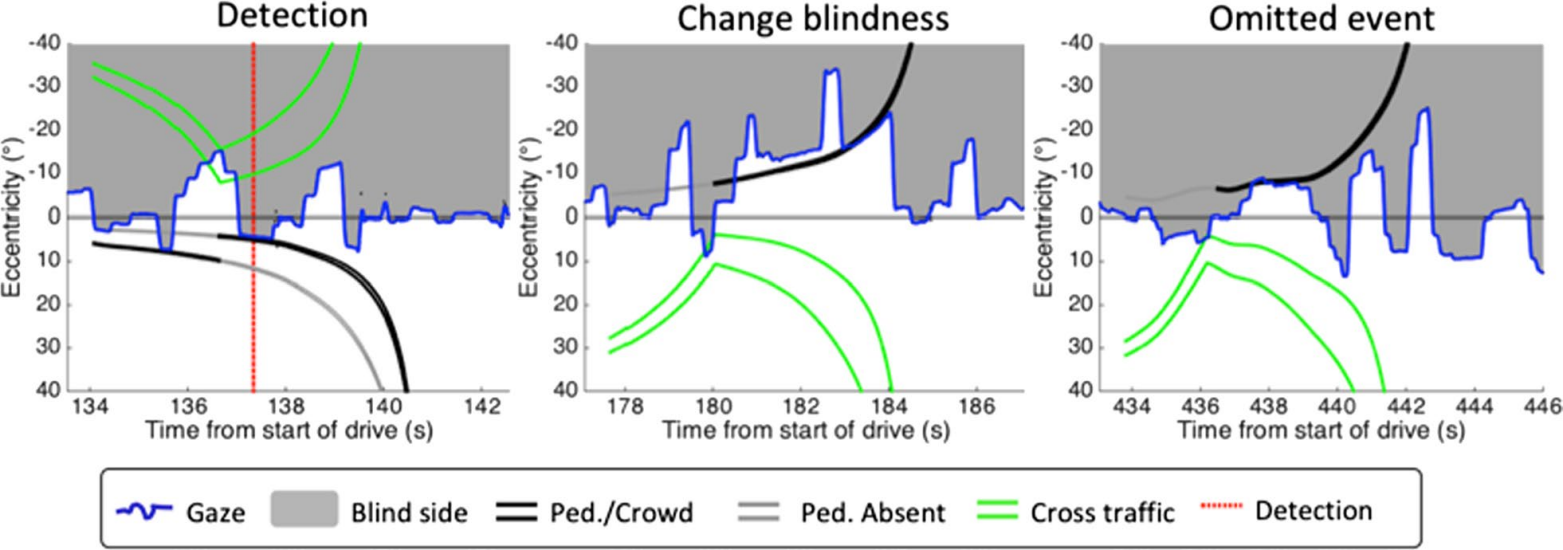
Examples of event data showing gaze (blue lines) and eccentricities of the critical pedestrian (black lines) and extent of the cross-traffic (green lines). In each example, an individual with left HVFL (gray area) has triggered the critical pedestrian. The pedestrian eccentricity prior to appearing is represented as a gray line. That location is visible in a seeing part (white area) of the visual field before and after the appearance in the left and middle panels, but not in the right panel. In the first example (left), representing Condition B, the individual looks at the cross-traffic, triggering the pedestrian far from the crosswalk to disappear and the appearance of the critical pedestrian near the crosswalk. The participant indicates that they detected the pedestrian appear (red dotted line). In the second example (middle), representing Condition A, the pedestrian is triggered, but there is no detection following sight of the pedestrian, and thus, this event was considered change blindness. In the last example (right), representing Condition A, the location of the pedestrian prior to the change was never visible. So, even though they triggered the pedestrian and failed to detect the change, this was not considered change blindness and was omitted from analyses

Next, we determined if the location of the change was ever in the seeing portion of the visual field (i.e., visible) both before and after the change was triggered (Fig. 4). If the change was triggered and the location was visible before and after the change, but the participant failed to press the indicator button following the change, then we categorized the event as “change blindness.” If they did press the response button after the change, then the event was categorized as “detection” (i.e., not change blindness). Events where the location of the change was in the blind hemifield either before or after the change, and therefore not perceived, were omitted from analysis because failing to detect the change in those situations would not be indicative of change blindness. All of these events were visually checked for accuracy, and no events were removed for data loss. Out of the 1188 possible events, 886 were events where gaze met the above criteria. Of these, 615 were triggered (494 gaze-triggered, 121 distance-triggered) and 74 of the triggered events were categorized as change blindness. For events that qualified and were categorized as “detection,” we calculated reaction time as the difference in time between when the pedestrian appeared and when the participant pressed the horn.

### Statistical analyses

#### Main analyses

Our main analyses concerned the number of triggered changes and the rates of change blindness between vision groups (HVFL vs. NV). The number of events triggered and number of valid change blindness events were analyzed with generalized linear mixed (GLM) effects models in MATLAB using *fitglme.m* (fitmethod: Laplace). Event number by scenario and participant ID were included as random effects with vision group allowed to vary between scenario and participant ID.

#### Secondary analyses

Our secondary analyses focused on evaluating factors that may have contributed to the change blindness rates found in the main analyses and on reaction time of detection of the change. First, we evaluated the side of the change (blind hemifield in HVFL, seeing hemifield in HVFL, and NV) as fixed factors. Then, we included condition (A vs. B vs. C) as a fixed factor in the HVFL vs NV model described in the main analyses section. Then, we examined the interaction between vision group and condition by comparing a model with the interaction term to a model without the interaction using a likelihood ratio test (*compare.m*). The same procedure was used to examine the effect of the change eccentricity on change blindness rate. The eccentricity of the change was calculated by taking the absolute value of the difference between the eccentricity of gaze and the change at the time of the change. Reaction time was log10 transformed to normalize the data and then analyzed using a linear mixed effect model with the same random effects described above. For models that did not converge, we used a simpler random effects structure by removing random slopes for the fixed factors. For the descriptive statistics in the results section, average values were calculated by taking the average across participants and standard error was calculated by dividing the standard deviation by the square root of the sample size. For reaction time, the log10 values were transformed back into seconds before taking the average and standard deviation across participants.

## Results

### Triggered events

**Fig. 5:**
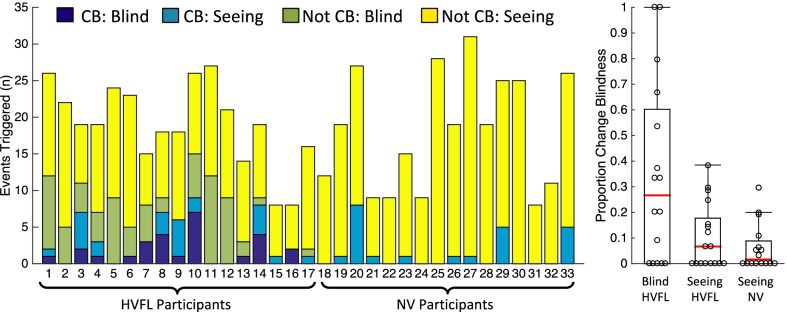
On the left, each bar represents a different participant with the color corresponding to the number of events per category. On the right, boxplots represent the proportion of change blindness events for HVFL and NV participants based on the hemifield in which the change appeared. Individual markers (open circles) in the figure on the right correspond to the proportion of events that were categorized as change blindness for each participant. CB = change blindness, Blind = events that appeared in the blind hemifield in those with HVFL, Seeing = events that appeared in the seeing hemifield of those with HVFL and NV

Results are displayed in Fig. 5. There was no significant effect of vision group (average HVFL = 71.8%, SEM = 4.4% vs. average NV = 65.5%, SEM = 6.2%) on the number of triggered events [*b* = 0.2, se = 0.5, *t* = 0.43, *p* = 0.67]. However, there were fewer triggered events that appeared in the blind hemifield of those with HVFL than their seeing hemifield (average = 52.9%, SEM = 7.1% vs average = 86.2%, SEM = 4.3%) [*b* = 2.7, se = 0.5, *t* = 5.6, *p* < 0.0001] and in the blind hemifield compared to those with NV [*b* = 1.2, se = 0.7, *t* = 0.6, *p* = 0.043]. In addition, there were significantly more triggered events in the seeing hemifield of participants with HVFL than NV [*b* = 1.5, se = 0.6, *t* = 2.4, *p* = 0.018].

### Change blindness events

There was a significant effect of vision group (average HVFL = 16.7%, SEM = 3.7% vs. average NV = 6.3%, SEM = 2.3%) on the number of change blindness events [*b* = 2.5, se = 0.8, *t* = 3.1, *p* = 0.002]. Those with HVFL had more change blindness events to changes that appeared in their blind hemifield (average = 34.6%, SEM = 8.9%) [*b* = 3.5, se = 0.8, *t* = 4.1, *p* < 0.001] and seeing hemifield (average = 10.4%, SEM = 2.5%) [*b* = 2.0, se = 0.9, *t* = 2.3, *p* = 0.023] than those with NV. Further, the secondary analysis suggested there was more change blindness to changes that appeared in the blind than seeing hemifield in those with HVFL [*b* = 1.5, se = 0.43, *t* = 3.5, *p* < 0.001].

### Effect of the different change conditions

There were no significant differences in the number of change blindness events between condition A and B [*b* = 0.26, se = 0.62, *t* = 0.4, *p* = 0.67], A and C [*b* = 1.2, se = 0.66, *t* = 1.9, *p* = 0.06], and B and C [*b* = 1.0, se = 0.57, *t* = 1.7, *p* = 0.08]. Further, there was no significant interaction between condition and side of the change [*χ*^2^(2) = 2.54, *p* = 0.28].

### Effect of the change eccentricity

There was no significant main effect of change eccentricity on change blindness rate [*b* = 0.01, se = 0.02, *t* = 0.5, *p* = 0.65] nor was there a significant interaction between eccentricity and the side of the change [*χ*^2^(1) = 0.44, *p* = 0.51].

### Reaction time

**Fig. 6:**
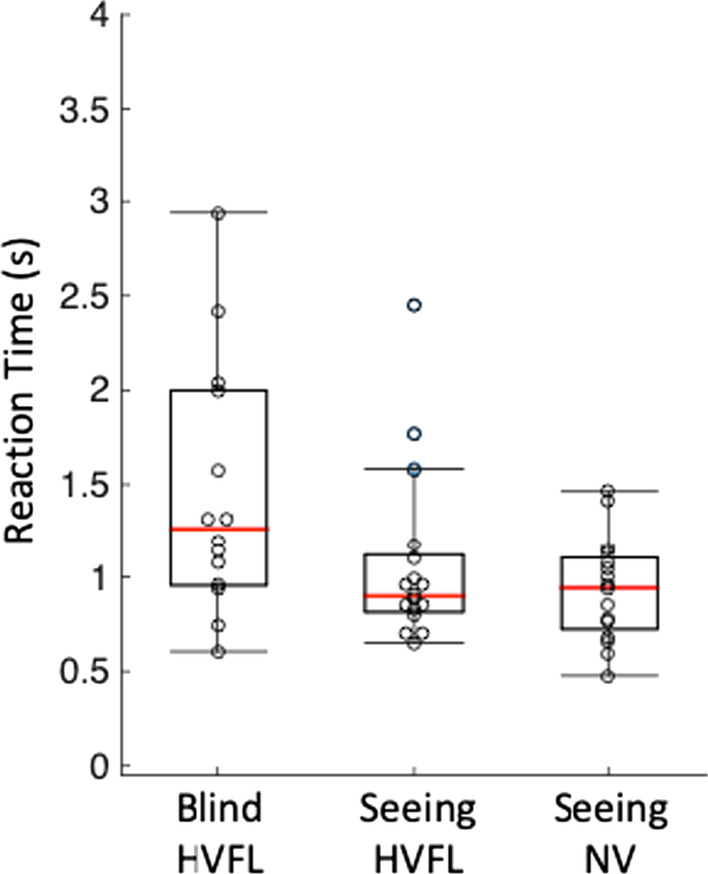
Boxplots of the median reaction time across participants for detections of the changes that appeared in the blind hemifield and seeing hemifield in those with HVFL and in those with NV. Individual markers (open circles) in the figure correspond to median RT for each participant

Reaction time data are displayed in Fig. 6. There was a significant effect of vision group (average HVFL 1.05 s, SEM = 0.10 s vs. average NV = 0.86 s, SEM = 0.06 s) on reaction time [*b* = − 0.09, se = 0.04, *t* = 2.01, *p* = 0.04]. For those with HVFL, detections were significantly slower for changes that occurred on the blind hemifield (average 1.44 s, SEM 0.17 s) than detections on the seeing hemifield (average 0.99, SEM 0.1 s) [*b* = 0.11, se = 0.04, *t* = 2.6, *p* = 0.009] and on the seeing side in those with NV [*b* = 0.17, se = 0.06, *t* = 3.1, *p* = 0.002]. There was no significant difference between the seeing hemifield in those with HVFL and those with NV [*b* = 0.06, se = 0.05, *t* = 1.33, *p* = 0.18].

## Discussion

We developed a novel change blindness paradigm in a driving simulator to evaluate change blindness in those with HVFL and in those with normal visual fields. Both groups of participants experienced change blindness to a pedestrian that appeared on the opposite side of where they were looking. The main finding was that those with HVFL experienced more change blindness than those with NV. A secondary analysis suggested that those with HVFL experienced more change blindness to changes that appeared in the blind hemifield than the seeing hemifield. Furthermore, those with HVFL had slower reaction times to changes on their blind hemifield than their seeing hemifield. And finally, there was no significant difference in reaction times to seeing side changes between those with HVFL and those with NV.

Interestingly, those with HVFL triggered significantly more changes to appear in their seeing than blind hemifield, likely because they could monitor the cross-traffic in peripheral vision when it approached from the seeing hemifield, thus not triggering the appearance of the pedestrian in the blind hemifield which was contingent upon a fixation on the cross-traffic. On the other hand, participants could not use peripheral vision to monitor cross-traffic approaching from the blind hemifield and would have to scan toward that side and fixate on the cross-traffic, hence triggering the appearance of the pedestrian in the seeing hemifield. This may reflect a compensatory strategy in those with HVFL, who tend to have more fixations and saccades toward the blind than seeing hemifield when driving (Bowers et al., 2014; Wood et al., 2011; Lövsund et al., 1991).

The results of this study corroborate other findings of impaired detection performance in individuals with HVFL (Zihl, 1995; Hardiess et al., 2010; Muir, 2017; Bowers et al., 2009; Swan et al., 2021; Bahnemann et al., 2015). Many of the deficits in target detection found in previous studies were the result of inadequate compensation (i.e., infrequent scans to the blind hemifield or insufficiently large scans toward the blind hemifield, e.g., Papageorgiou et al., 2012). Here, our results suggest that even with adequate compensation (the majority of critical pedestrians were fixated), individuals with HVFL may still fail to detect potential hazards in the blind hemifield and may also fail to detect potential hazards in the seeing hemifield.

There were wide individual differences in change blindness rates in both vision groups (ranging from 0 to 42% in those with HVFL and 0% to 30% in those with NV). These wide individual differences are a characteristic of the literature on detection performance of those with HVFL (e.g., see review Bowers, 2016). Many studies have examined participant characteristics that could predict detection performance in those with HVFL, such as duration or side of visual field loss (Bowers et al., 2009; Papageorgiou et al., 2012; Alberti et al., 2017). One of the few characteristics that have predicted blind side detection performance was age (Bowers et al., 2009; Papageorgiou et al., 2012; Alberti et al., 2017). Similarly, change blindness rates do tend to increase with age (e.g., Rizzo et al., 2009). However, larger samples than those in the current study would be needed to elucidate individual differences that could predict change blindness performance.

Neither change condition nor the eccentricity of the change significantly affected change blindness rate. Our critical intersections contained only the pedestrian of interest and sometimes the crowd of pedestrians, and it is possible that participants quickly learned where to look for changes, which means that the other pedestrians (i.e., the crowds) did not distract participants. Despite this, some participants still had difficulty remembering whether a change in location occurred, as indicated by the increased change blindness rates in those with HVFL. Our results fall more in line with “looked-but-failed-to-see” incidents that arise not from a perceptual failure, but from a failure during processing (Koustanaï et al., 2008). Increased mental (Pérez-Moreno et al., 2011) and perceptual (Murphy & Greene, 2016) load have been shown to both increase rates of inattentional blindness. If the task were more difficult, e.g., if pedestrians were present on both sides and if their changed position were less predictable, then the number of pedestrians and perhaps their eccentricity to the intersection may have increased change blindness rates, which may result in failing to perceive the critical changing pedestrian.

Finding change blindness in the seeing hemifield in both vision groups corroborates previous research investigating change blindness in NV participants in driving situations using flicker paradigms (Beanland et al., 2017; Caird et al., 2005; Galpin et al., 2009; Koustanaï et al., 2012; McCarley et al., 2001, 2004), while watching videos (Martens, 2011), and while driving in the simulator (Harms & Brookhuis, 2016; Charlton & Starkey, 2013; Zheng et al., 2010; Velichkovsky et al., 2002; Lee et al., 2007; Filtness, et al., 2020). Importantly, we demonstrated change blindness to a potential hazard within a traffic scene without requiring an artificial visual disruption (see also Velichkovsky et al., 2002). While the changes represented a potentially hazardous situation, they were by themselves not hazardous, which is a limitation of the paradigm. Using gaze to trigger changes could be used to further understand the impacts of other factors that may increase rates of failures of visual awareness while driving, such as distraction (e.g., Hyman et al., 2010; McCarley et al., 2004; Strayer et al., 2003), age (Caird et al., 2005; Rizzo et al., 2009), or when the hazards do not meet the attentional set as determined by the goals of the driver (Most & Astur, 2007; Murphy & Greene, 2016). While those with HVFL failed to detect more changes than those with NV, they still fixated on the pedestrian approximately 97.7% of the time, compared to 100% in those with NV. Thus, the direct implications of change blindness and failures to detect hazards need to continue to be investigated.

One limitation is that the majority of our participants with HVFL were not current drivers. The study was conducted in Massachusetts, where individuals with HVFL are not legally able to drive because the minimum horizontal visual field extent required for licensure is 120°. While all participants completed practice drives prior to the experimental drives, those who had ceased driving may have experienced greater difficulty driving in the simulator which could have contributed to higher change blindness rates.

## Conclusion

We developed a driving simulator paradigm to investigate change blindness to a potential hazard that suddenly appears while looking at the opposite side of the intersection. We predicted and found that individuals with a HVFL experienced more change blindness than those with normal visual fields. These results corroborate other findings regarding impaired detection performance in those with HVFL and suggest that even with sufficient compensation via scanning, individuals with HVFL may still experience failures of visual awareness to objects in their blind hemifield. Similar to other findings in the HVFL literature, there were wide individual differences, with some participants experiencing no change blindness and others failing to detect more than a third of events. Finding change blindness to changes in the seeing hemifield in those with HVFL and in those with NV suggests that some hazard detection failures when driving may be veridical failures of visual awareness.

## Data Availability

The datasets used and/or analyzed during the current study are available from the corresponding author on reasonable request.

## Abbreviations

LBFTS: “Looked but failed to see”
HVFL: Homonymous visual field loss
NV: Normal vision
SEM: Standard error of the mean
GLM: General linear model
GPS: Global positioning system
